# Predicting homelessness: Housing risk insights from latent class analysis

**DOI:** 10.1371/journal.pone.0306534

**Published:** 2024-07-05

**Authors:** Katherine E. Marçal, Nicholas Barr

**Affiliations:** 1 School of Social Work, Rutgers University, New Brunswick, NJ, United States of America; 2 School of Social Work, University of Nevada Las Vegas, Las Vegas, NV, United States of America; Hunan University, CHINA

## Abstract

Millions of families with children in the U.S. struggle to afford adequate housing. Housing cost burden places families at risk for homelessness, and prevention efforts are hindered by limited understanding of insecure housing experiences at the margins. The present study investigated variation in housing insecurity experiences in a sample of mothers, as well as which risk profiles were most strongly associated with subsequent homelessness. Latent class analysis identified four distinct subgroups of housing insecurity: “Stable,” “Unstable,” “Rent-Focused,” and “Strategic Bill-Paying.” Classes differed on whether they made rent or utility payments on time, experienced utility shutoffs, or were evicted. Mothers who missed rent payments were significantly more likely to experience subsequent homelessness, whereas those who prioritized rent were more likely to have their utilities shut off but remain housed. Policy efforts should emphasize increased wages, rent control, changes to zoning laws and tax codes to prioritize affordable housing, and benefits that help mothers maintain their incomes such as comprehensive healthcare, paid maternity leave, and subsidized childcare.

## Introduction

Homelessness is difficult to predict, impeding effective prevention [1–3]. Nearly all families who become homeless are poor, but very few poor families become homeless [4]. Homelessness often follows periods of unaffordable, precarious, and chaotic housing situations [5, 6], but little empirical evidence informs which constellations of housing experiences translate to increased homelessness risk. Scarce prevention resources necessitate improved ability to identify households at risk and their unique pathways to homelessness.

### Family homelessness

Families with children comprise one-third of the U.S. homeless population. One in six people experiencing homelessness are under age 18 [7], and most do so as part of family households. These households are overwhelmingly female-headed, young, and poor [4, 8] and face ongoing barriers to housing and economic stability [9]. Limited affordable housing along with stagnant wages that fail to keep pace with inflation place low-income families at constant risk for losing their homes. Children exposed to homelessness display a number of adverse outcomes including mental health and behavior problems [10, 11], poor academic performance [12, 13], and worse physical health compared to their poor but housed peers [14].

Definitions of homelessness vary, but emphasize the chaotic, destabilizing, vulnerable nature of lacking safe, stable, affordable housing [15]. The U.S. Department of Housing and Urban Development (HUD), which funds most homeless services in communities across the country, considers families to be experiencing “literal homelessness” if they are staying in a shelter, vehicle, abandoned building, outside, or some other place not meant for human habitation [16]. Under this definition, an estimated half a million people in families with children experience literal homelessness each year [4, 17]. The U.S. Department of Education further includes families doubled up with others due to loss of housing or economic hardship in its definition of homelessness according to the McKinney–Vento Homeless Assistance Act, which mandates homeless service delivery in public schools (42 U.S.C. § 11434a); an estimated one million children experience doubling up homelessness [18].

### Family housing insecurity

Meeting the criteria of official homelessness definitions enables families to access funded services. However, homelessness is a relatively rare occurrence for low-income, at-risk families. Over 5 million households with children experience poverty each year in the United States, whereas fewer than 1.5 million experience homelessness or doubling up according to HUD and McKinney-Vento definitions [4, 8, 18]. Thus, programs targeting these families miss the many millions who experience other forms of housing hardship such as cost burden or eviction in the face of affordable housing shortages throughout the country. Failure to address insecurity that does not meet official homelessness definitions risks worsening housing problems over time.

Far more prevalent than homelessness is housing insecurity, which encompasses a broader range of experiences that may portend risk for future homelessness [8]. Even prior to the COVID-19 pandemic, affordable rental housing supply was extremely limited for the lowest-income households [19], forcing many to live in unstable or unaffordable accommodations [9]. Nearly 8 million U.S. households were identified as having extremely low incomes relative to rent prices or being severely cost-burdened by housing in 2019 [6], and an estimated 3.6 million evictions are filed each year [20]. The economic upheaval brought on by the pandemic has only intensified scarcity of affordable housing options for low-income families [21]. Additional, less visible forms of housing insecurity include frequent moves and missing other bills to make rent [9, 22]. Housing insecurity thus takes many forms, but challenges identification and intervention because many HUD-funded services remain unavailable until families lose their homes entirely [1].

Limited affordable housing options force families to make tradeoffs between domains of housing such as quality, stability, location, and affordability [23–25]. Inadequate income to cover basic needs forces families to weigh making rent payments versus paying for other necessities such as utilities, groceries, and childcare [26]. Scarcity increases stress [27, 28] and impacts decision-making [29, 30]. Disparate housing experiences may therefore reflect disparate strategies for navigating a tight rental market with scarce resources, but little research investigates these dynamics. Furthermore, the relationship between various strategies and long-term housing outcomes is not well understood. Limited knowledge of various constellations of housing risk factors impede ability to identify the most vulnerable households, better target services and supports, and prevent subsequent homelessness.

### A person-centered approach to housing insecurity

Because experiences of housing insecurity vary so widely, traditional variable-centered approaches that assume parameters can be averaged across individuals may overlook important complexity [31]. Person-centered approaches, in contrast, consider subgroups of individuals who vary across parameters in meaningful ways [32]. Housing insecurity manifests in a variety of ways that may reflect families’ default or “go-to” strategies for responding to financial hardship; households with relatives and friends nearby may seek to borrow money or double up, whereas households with fewer social connections may take on debt or forgo other basic needs to afford housing. Housing problems may also cluster together given specific household risk factors and decision-making; for example, missed rent payments may co-occur with evictions, whereas missed utility payments may occur with shutoffs. Little empirical evidence informs how these strategies cluster together, and how they relate with subsequent homelessness risk.

Person-centered mixture modeling approaches like LCA facilitate data clustering and statistical inferences by assigning individuals within a heterogeneous population to homogenous subgroups with distinct patterns of responses on categorical variables [33]. LCA is valuable where variable centered analyses, which assume participants are part of a single population, may obscure the presence of heterogenous subgroups with differences on key indicators and their class-based associations with distal outcomes [34]. In the context of complex, chaotic, and nuanced experiences like housing instability and cost-burden, a person-centered approach may uncover heterogeneity critical to efficient homelessness prevention efforts.

### Present study

The present study aimed to investigate characteristics of families experiencing various types of housing insecurity, and what housing experiences indicated increased risk for subsequent homelessness. Specifically, we tested the following research questions: 1) Do subtypes of housing insecurity exist in a sample of at-risk mothers? 2) Which characteristics distinguish housing insecurity subtype? 3) Do housing insecurity subtypes distinguish homelessness risk? Findings will inform efforts to identify families experiencing housing problems and potential leverage points for homelessness prevention.

## Methods

### Data and participants

Data for the present study came from the Future of Families (formerly “Fragile Families”) and Child Well-Being Study (hereafter FFCW). FFCW used a cluster stratified random sampling strategy to select 20 large American cities, hospitals within cities, and mothers within hospitals who had recently given birth; unmarried mothers were intentionally oversampled, resulting in a final sample that skewed low-income and minority relative to the general U.S. population [35]. Baseline data were collected 1998–2000, with follow-up interviews occurring at 1-, 3-, 5-, 9-, and 15-year intervals.

The original FFCW study was approved by the Princeton University Institutional Review Board (IRB) as well as approval from local hospital IRBs. The present study utilized the de-identified FFCW Public Use data accessed January 17, 2019 and thus did not require additional IRB approval. The analytic sample was limited to mothers who retained at least partial custody of children at the Year 9 and 15 interviews, when children were approximately nine and 15 years old (*N* = 2,875; Table 1).

**Table 1:** Sample description.

	M (SD)	N (%)	% Imputed
Race			0.21%
White		608 (21.15%)	
Black		1,450 (50.44%)	
Hispanic		711 (24.73%)	
Other		106 (3.69%)	
Age	25.26 (6.04)		0.07%
Highest Education			1.15%
Less than high school degree		575 (20.00%)	
High school degree/GED		614 (21.36%)	
Some college		1,226 (42.64%)	
College degree		460 (16.00%)	
Household Size	4.75 (1.62)		1.25%
Depression		515 (17.91%)	0.17%
Married and/or Cohabitating		1,732 (60.24%)	1.32%
Household Income	$46,868 ($51,050)		1.25%
Employed		1,853 (64.45%)	1.25%
Welfare/TANF Receipt		319 (11.10%)	1.25%
Housing Insecurity (Year 9)			
Missed rent/mortgage payment		536 (18.64%)	1.25%
Missed utility payment		904 (31.44%)	1.32%
Electricity shut off		289 (10.05%)	1.25%
Borrowed money		880 (30.61%)	1.29%
Evicted		73 (2.54%)	1.25%
Homeless (Year 15)		148 (5.15%)	0.17%

The sample was majority nonwhite. Half of mothers were Black (50.4%), one in four were Hispanic/Latinx 24.7%), and a small portion were some other race (3.7%). Only one in six (16.0%) mothers had completed a college degree by Year 9, whereas one in five (20.0%) had not completed high school. The majority of mothers were married and/or cohabitating with a partner (60.2%), two-thirds of mothers were employed, and 18% screened positive for major depressive disorder at the Year 9 interview.

The most common experiences of housing insecurity at Year 9 were missed utility payments (31.4%) and borrowing money (30.6%). The least common were eviction (2.5%) and electricity shutoffs (10.1%). Homelessness at Year 15 was rare, affecting just 5.2% of mothers.

### Measures

#### Latent class indicators

Latent subgroups of *housing insecurity* were determined using five dichotomous indicator variables assessing whether or not mothers reported certain housing experiences in the past 12 months collected at the Year 9 interview. *Missed rent/mortgage* indicated whether or not mothers reported having missed a rent or mortgage payment, *missed utility* indicated a missed utility (electric, gas, etc.) payment, *utility shutoff* indicated whether utilities had been shut off due to delinquent payment, *borrowed money* indicated whether mothers had borrowed money from friends or family to pay bills, and *evicted* indicated whether mothers had been evicted for nonpayment of rent in the past 12 months.

#### Distal outcome

*Homelessness* was assessed at the Year 15 interview and corresponded to a combination of the HUD and McKinney Vento Act federal definitions of homelessness for families with children, which include “literal homelessness” (living in shelter or on the streets; Homeless Emergency Assistance and Rapid Transition to Housing (HEARTH) Act) [16], as well as “doubling up” (moving in with others due to inability to afford housing) [36]. Mothers reported whether or not they had spent a single night in a shelter, vehicle, abandoned building, on the streets, or somewhere else not meant for human habitation, and whether they had moved in with others due to inability to afford housing since the previous interview.

#### Predictors

A number of predictors were used to determine latent class membership. Mothers’ *race/ethnicity* was dummy coded as White (reference), Black, Hispanic, and Other. *Age* indicated the mother’s age in years at the study focal child’s birth. *Education* categorized mothers according to their highest level of education completed (less than a high school degree, high school degree/GED, some college, college degree or higher) by the Year 9 interview. *Household size* indicated the number of people, including the mother and study focal child, living in the household at Year 9. *Depression* was assessed at Year 9 using a series of questions about mothers’ duration and intensity of symptoms such as crying, feeling sad, loss of appetite, and low energy; mothers were classified as probable cases or non-cases for major depressive disorder consistent with the Diagnostic and Statistical Manual of Mental Disorders fourth edition (DSM-IV) using the World Health Organization’s Composite International Diagnostic Interview–Short Form (CIDI-SF) [37]. Also at Year 9, mothers reported whether they were currently *married to and/or cohabitating* with an intimate partner, their current household income, whether or not they were currently *employed*, and whether they received any welfare or income *transfers* from programs such as Temporary Assistance to Needy Families (TANF) or Supplemental Security Income (SSI).

### Analytic strategy

Statistical analyses were conducted in three phases. First, we specified and fitted a series of LCA models with *k*+1 classes added at each iteration to identify the best fitting class structure for year 9 class indicator variables. Second, we used the 3-step approach to auxiliary variable modeling in LCA described by Asparouhov and Muthén [38], where modal class assignment places individuals into the class for which they have the highest post-estimation probability, to fit a multinomial logistic regression model with classes as dependent variables and year 9 demographic, depression, income, and employment as independent variables. Finally, we applied Lanza and colleagues’ [39] model-based approach to distal outcomes, where the categorical distal outcome is included in the class estimation model as an auxiliary variable, to produce probabilities of year 15 homelessness by class and odds ratios expressing the relative odds of homelessness by class. This method is optimal with categorical distal outcomes because it prevents the distal outcome variable from impacting class membership for individual observations [39].

To identify the best class solution for these data, LCA models with 1 to 5 classes were compared across fit indices including Akaike information criteria (AIC), Bayesian information criteria (BIC), sample size adjusted Bayesian information criteria (ssBIC), and entropy (E). We also applied the parametric bootstrapped likelihood ratio (BLRT) test to examine the null hypothesis that each *k-*class model was sufficient to describe the sample compared to the *k* + 1 class model. Finally, class solutions were evaluated for explanatory utility and interpretability. For AIC, BIC, and ssBIC values, lower numbers indicate better fit, while higher E values scaled from 0 to 1 are indicative of better separation between classes.

## Results

**Fig 1:**
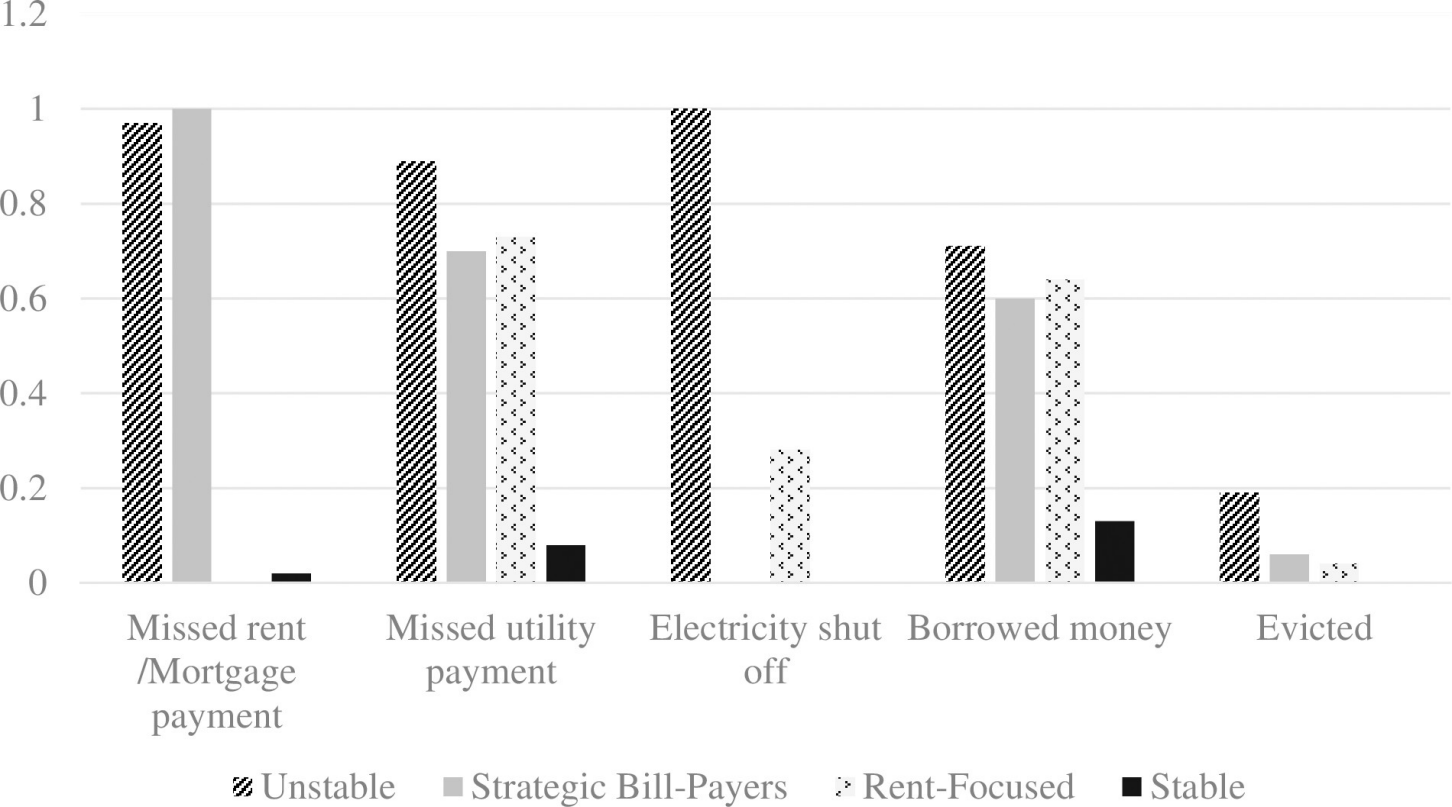
Item response probabilities for the 4-class solution.

Phase one analyses suggested that a 4-class solution provided the best fit to the data (Table 3). Fit index values across 1–5 class models are shown in Table 2. AIC and model entropy supported the 4-class over the 3-class model, while BIC and ssBIC favored the 3-class model. Results of the BLRT test supported the 4- over the 3-class model (H0 LL = -5492.113, p<0.05). Class meaning and interpretability were also improved in the 4-class model, which distinguished between rent-focused and strategic bill paying classes that were collapsed in the 3-class model. A 5-class solution was specified but rejected due to extremely small (<2%) class sizes, higher AIC, BIC, and ssBIC values than the 4-class model, and a nonsignificant BLRT test statistic comparing 4-and 5-class models. Fig 1 provides a plot of model estimated probabilities for class indicator variables.

**Table 2:** Model fit indices across 1- through 5-class solutions.

Model	Log Likelihood	AIC	BIC	ssBIC	Entropy
1 Class	-6221.828	12453.66	12483.48	12467.59	NA
2 Class	-5506.610	11035.22	11100.82	11065.87	0.720
3 Class	-5492.113	11018.23	11119.61	11065.60	0.760
4 Class	-5483.724	11013.45	11150.62	11077.54	0.840
5 Class	-5481.329	11020.66	11193.61	11101.47	0.847

The largest class, “Stable,” comprised about 65% of the sample and was characterized by very low probability values across all class indicators. This class displayed a less than *p* = 0.10 probability of experiencing all housing insecurity types with the exception of borrowing money (*p* = 0.13). The second class, “Rent-Focused,” comprised about 17% of the sample and had very low probability of missing rent or eviction but comparatively high probabilities of utility shut-off and borrowing money. The third class, “Strategic Bill-Paying,” comprised about 12% of the sample and had high probabilities of missing rent and utility payments and borrowing money but a very low probability of utility shut-off and eviction. The fourth and smallest class, “Unstable,” comprised about 5% of the sample and was characterized by a high probability of missing rent and the highest probabilities of missing utility payments, having utilities shut off, borrowing money, and eviction.

Results of the multinomial logistic regression analyses conducted in phase two estimated which predictors were associated with class membership relative to the “Stable” class (Table 3).

**Table 3:** Multinomial logistic regressions predicting latent class membership.

	Rent-Focused	Strategic Bill-Paying	Unstable
	vs. Stable	vs. Stable	vs. Stable
Race *(ref = White)*			
Black	0.95 (0.63, 1.45)	1.10 (0.76, 1.61)	0.69 (0.41, 1.15)
Hispanic	**0.43 (0.26, 0.72)**	**0.64 (0.41, 0.99)**	0.70 (0.40, 1.23)
Other	0.93 (0.35, 2.50)	0.87 (0.36, 2.13)	1.07 (0.38, 3.07)
Age	0.98 (0.95, 1.01)	0.98 (0.96, 1.01)	0.98 (0.95, 1.01)
Highest Education *(ref = Less than high school degree)*			
High school degree/GED	**1.67 (1.07, 2.60)**	**2.10 (1.38, 3.21)**	**1.11 (1.05, 1.29)**
Some college	1.32 (0.87, 2.01)	**1.95 (1.32, 2.88)**	1.44 (0.90, 2.30)
College degree	0.71 (0.34, 1.47)	1.70 (0.97, 3.00)	1.11 (0.50, 2.43)
Household Size	1.09 (0.99, 1.20)	1.05 (0.97, 1.14)	**1.16 (1.05, 1.29)**
Depression	**1.89 (1.30, 2.75)**	**1.87 (1.36, 2.57)**	**2.64 (1.77, 3.94)**
Married and/or Cohabitating	1.09 (0.77, 1.54)	1.05 (0.78, 1.41)	0.74 (0.50, 1.10)
Household Income	**0.98 (0.97, 0.98)**	**0.98 (0.98, 0.99)**	**0.98 (0.97, 0.99)**
Employed	1.10 (0.79, 1.54)	1.17 (0.87, 1.57)	1.49 (0.99, 2.22)
Welfare/TANF Receipt	0.89 (0.56, 1.42)	1.08 (0.73, 1.60)	1.54 (0.94, 2.53)

*Note*: Bold indicates statistical significance at the p < 0.05 level

Having a high school degree or GED (OR = 1.67, 95% CI: 1.05, 2.60) and depression (OR = 1.89, 95% CI: 1.30, 2.75) were positively associated with being “Rent-Focused” while Hispanic ethnicity (OR = 0.43, 95% CI: 0.26, 0.72) and income (OR = 0.89, 95% CI: 0.97, 0.98) were negatively associated. Household size (OR = 1.16, 95% CI: 1.05, 1.23) and depression (OR = 2.64, 95% CI: 1.77, 3.93) were positively associated with membership in the “Unstable” class relative to “Stable,” while income (OR = 0.98, 95% CI: 0.97, 0.99) was negatively associated. Having a high school degree or GED (OR = 2.10, 95% CI: 1.38, 3.21), having some college experience (OR = 1.95, 95% CI: 1.32, 2.88), and depression (OR = 1.87, 95% CI: 1.36, 2.57) were positively associated with being in the “Strategic Bill-Paying” class, while Hispanic ethnicity (OR = 0.64, 95% CI: 0.41, 0.99) and income from transfers (OR = 0.98, 95% CI: 0.98, 0.99) were negatively associated.

Results of phase three equality tests of means and probabilities for Year 15 homelessness across classes are shown in Table 4. The “Stable” class had the lowest probability of Year 15 homelessness (0.03), while the “Unstable” class had the highest probability (0.15); belonging to the “Unstable” class at Year 9 was associated with a six-fold increased risk for homelessness at Year 15 (χ^2^ = 17.0, p < 0.001; Table 5). Both the “Rent-Focused” and “Strategic Bill-Paying” classes were associated with significant higher probabilities of homelessness relative to the “Stable” class (χ^2^ = 16.4, p < 0.001 and χ^2^ = 9.4, p < 0.01, respectively), but did not display significantly differing risks from each other (Table 6).

**Table 4:** Item response probabilities and bootstrapped 95% confidence intervals by class.

	Missed rent / mortgage payment	Missed utility payment	Electricity shut off	Borrowed money	Evicted
Class	P (bootstrapped 95% CI)				
Unstable	0.97(0.77, 1.00)	0.88(0.79, 0.94)	1.00 (1.00, 1.00)	0.71(0.61, 0.78)	0.19(0.12, 0.25)
Strategic bill payer	1.00(1.00, 1.00)	0.71(0.62, 0.76)	0.00 (0.00, 0.00)	0.60(0.51, 0.64)	0.06(0.03, 0.09)
Rent-focused	0.00(0.00, 0.00)	0.73(0.63, 0.81)	0.28(0.17, 0.34)	0.64(0.52, 0.75)	0.04(0.02, 0.06)
Stable	0.02(0.00, 0.03)	0.08(0.05, 0.11)	0.00(0.00, 0.01)	0.13(0.11, 0.15)	0.00(0.00, 0.01)

**Table 5:** Probabilities and odds ratios by class for outcome homelessness at year 15.

Class	Probability	S.E.	OR	95% CI
Stable	0.03	0.01	-	-
Rent-Focused	0.08	0.01	2.86	1.63, 5.01
Strategic Bill-Paying	0.07	0.02	3.66	2.24, 5.98
Unstable	0.15	0.03	6.23	3.58, 10.92

*Note*: Reference class is Stable.

**Table 6:** Head-to- head differences tests by class for outcome homelessness at year 15.

Test	*X* ^*2*^	p-value
Overall test	**37.00**	**<0.001**
Unstable vs. Strategic	2.85	0.09
Unstable vs. Rent-Focused	**5.37**	**<0.05**
Unstable vs. Stable	**16.95**	**<0.001**
Strategic vs. Rent-Focused	0.84	0.36
Strategic vs. Stable	**16.38**	**<0.001**
Rent-Focused vs. Stable	**9.35**	**<0.01**

*Note*: Bold indicates statistical significance at the p < 0.05 level

## Discussion

The present study investigated unique constellations of housing insecurity and their implications for future homelessness risk. Specifically, we identified subgroups of housing insecurity among vulnerable maternal households with children, characteristics associated with those subgroups, and subsequent homelessness risk in order to better design and target supports. Analyses identified four subgroups of housing insecurity in a sample of vulnerable mothers in 20 large American cities. Subgroup membership was distinguished by household size, maternal depression, and household income, and was associated with varying risk for subsequent homelessness.

Given the high-risk nature of the full sample, all four classes displayed some level of cost-burden through borrowing money to pay bills or missing utility payments. The strongest determinants of class separation were whether families missed rent payments or had their utilities shut off, suggesting that families made calculations about which bills to pay each month given their individual circumstances and perceptions of associated risks. The largest class, comprising two-thirds of the sample, displayed low levels of housing insecurity; this “Stable” class paid rent on time and avoided utility shutoffs and eviction. The smallest class, “Unstable,” struggled to afford basic needs despite borrowing money, with elevated rates of utility shutoffs and evictions. Two intermediate classes displayed alternate approaches to coping with financial burden; the first prioritized rent payments at all costs, leading to higher levels of missed utility payments and shutoffs but low levels of eviction. The second missed both rent and utility payments at high rates but were unlikely to experience either utility shutoffs *or* eviction, suggesting payments may have been timed strategically to minimize adverse outcomes–for example, mothers in this class may have alternated missing payments, or only skipped/delayed rent after discussing options with landlords. Both the “Rent-Focused” and “Strategic Bill-Paying” classes were characterized by similar probabilities of borrowing money and similarly low probabilities of eviction.

Several maternal and household-level characteristics point to differential risk for housing hardship. Unintuitively, mothers with a high school diploma experienced lower likelihood of belonging to the “Stable” class than mothers who had not completed high school; while lower education levels tend to point to greater socioeconomic hardship, it is possible that mothers who had not completed high school were younger on average and thus receiving family support. Compared to White mothers, Hispanic mothers were more likely to belong to the “Stable” class than either “Rent-Focused” or “Strategic Bill-Paying.” Hispanic/Latinx families may experience the protective benefits of *familismo*, a core cultural value that emphasizes supportive ties with immediate and extended kin [40]; Hispanic mothers in the sample may therefore have likewise benefited from family support that protected them from housing hardship. Maternal depression was associated with increased likelihood of belonging to the “Unstable,” “Rent-Focused,” or “Strategic Bill-Paying” class relative to the “Stable” class. Characterized by low mood and energy, depression can impede mothers’ abilities to engage in flexible decision-making required to navigate scarce resources in the low-income rental housing market [41–43]. Unsurprisingly, household income was protective against housing problems; higher income better enables families to afford basic needs with fewer sacrifices.

The “Unstable,” “Rent-Focused,” and “Strategic Bill-Paying” classes displayed significantly elevated risks for homelessness relative to the “Stable” class. “Rent-Focused” and “Strategic Bill-Paying” mothers did not differ from each other in terms of risk for subsequent homelessness, but “Rent-Focused” mothers were significantly less likely to experience subsequent homelessness compared to the “Unstable” class. Thus, consistent rent payments–sometimes at the expense of paying other bills–was in part associated with reduced homelessness risk over time. “Strategic Bill-Paying” families were no less likely to experience subsequent homelessness than “Unstable” families, but the former experienced less housing insecurity in the interim, indicating that while this type of calculated resource allocation could delay homelessness, creative budgeting could not overcome fundamental resource scarcity.

The different subgroups identified in the present study thus point to different strategies in coping with inadequate resources. While eviction and homelessness are common consequences of missed rent payments, shutoffs are a logical consequence of unpaid utility bills [26]. Families with inadequate resources to cover all household expenses must determine which bills get paid each month, and thus which consequences to risk. Utility shutoffs may be less immediately catastrophic than losing one’s home, but health conditions or extreme weather can make loss of water, heat, or air-conditioning dangerous depending on family circumstances. Eviction, conversely, may put families on the streets or in shelters, and can impact future ability to find housing. When household incomes are too low to cover basic needs, families must navigate scarcity faced with only bad options.

Findings underscore the unsustainable nature of the low-income rental housing market. The three non-“Stable” classes identified in the present study demonstrate differing strategies for managing unaffordable housing while the underlying issue remains inadequate income to afford housing costs. Nearly one in five mothers could not afford housing costs consistently. Unpaid rent is overwhelmingly the most commonly cited reason for tenant eviction [20, 44] and homelessness [8], and indeed the two classes mostly likely to report missed rent payments in the present study also displayed the highest levels of eviction and homelessness. Neither employment nor welfare receipt had any association with housing insecurity after controlling for total household income from all sources. Job training programs and work requirements for welfare likely offer no major benefit to reducing housing insecurity if housing costs continue to outstrip wages for low-income families [45]. Results support the view that the most impactful approach to reducing housing insecurity and homelessness is increasing wages, reducing housing costs, and offering rental assistance such that families can afford their monthly payments.

The present study highlights the underlying driver of homelessness as a failure of incomes to keep pace with housing costs for the lowest-wage earners. Interventions must contend with structural conditions and market mechanisms that make housing disproportionately scarce and expensive for low-income people. Given that nearly one in three mothers in the present study missed utility payments, utility assistance programs may reduce cost burden as well as decision-fatigue associated with juggling expenses for low-income families–particularly as extreme heat intensifies in tight rental markets across the American West, increasing the costs of utilities and consequences of shutoffs. Policy efforts should emphasize increased wages, rent stabilization and caps on arbitrary rent increases, changes to zoning laws and tax codes to prioritize construction of affordable housing [46], and benefits that help mothers maintain their incomes such as comprehensive healthcare, paid maternity leave, and subsidized childcare [47]. Fundamentally, policymakers may need to choose one of two core approaches to housing in stressed markets: preserving housing as an asset class whose purpose is to generate wealth for landlords and investors, or prioritizing affordable housing as a right for families whose labor provides the connective tissue of American cities and communities.

Findings must be contextualized by study limitations. FFCW exclusively sampled families from large U.S. cities, and the present analyses focused on data collected from later waves; findings cannot therefore be generalized to non-urban populations or households with young children, who may display different patterns of housing insecurity and homelessness [48]. Second, the study did not distinguish between homeowners and renters; although the vast majority of the sample were renters and eviction is generally a low-prevalence event, the presence of owners in the sample may explain in part the low levels of eviction. Third, no standard definition or measure of housing insecurity exists, and the items used in the present study may not cover the full range of potential indicators [9]. Finally, the present study did not account for shifts in housing profiles over time; rather, analyses were focused on how indicators of housing insecurity predicted subsequent homelessness. Future research should engage additional approaches such as latent transition analysis to investigate whether housing risk subtypes are stable over time.

## Conclusion

Despite limitations, the present study illuminates important variation in housing insecurity experiences and homelessness risk. Families deploy different strategies for coping with scarcity that may lead to varying short- and long-term housing experiences. Failure to address lack of affordable housing and supports for mothers struggling to meet basic needs further entrenches housing disparities and homelessness.
